# The Ability of Airborne Microalgae and Cyanobacteria to Survive and Transfer the Carcinogenic Benzo(a)pyrene in Coastal Regions

**DOI:** 10.3390/cells12071073

**Published:** 2023-04-02

**Authors:** Kinga A. Wiśniewska, Anita U. Lewandowska, Sylwia Śliwińska-Wilczewska, Marta Staniszewska, Gracjana Budzałek

**Affiliations:** 1 Institute of Oceanography, Department of Chemical Oceanography and Marine Geology, University of Gdansk, Av. M. Piłsudskiego 46, 81-378 Gdynia, Poland; 2 Department of Biology, Mount Allison University, 62 York St., Sackville, NB E4L 1E2, Canada; 3 Institute of Oceanography, Division of Marine Ecosystems Functioning, University of Gdansk, Al. M. Piłsudskiego 46, 81-378 Gdynia, Poland

**Keywords:** bioaerosols, airborne cyanobacteria, airborne microalgae, benzo(a)pyrene, PAHs

## Abstract

Air pollution has been a significant problem threatening human health for years. One commonly reported air pollutant is benzo(a)pyrene, a dangerous compound with carcinogenic properties. Values which exceed normative values for benzo(a)pyrene concentration in the air are often noted in many regions of the world. Studies on the worldwide spread of COVID-19 since 2020, as well as avian flu, measles, and SARS, have proven that viruses and bacteria are more dangerous to human health when they occur in polluted air. Regarding cyanobacteria and microalgae, little is known about their relationship with benzo(a)pyrene. The question is whether these microorganisms can pose a threat when present in poor quality air. We initially assessed whether cyanobacteria and microalgae isolated from the atmosphere are sensitive to changes in PAH concentrations and whether they can accumulate or degrade PAHs. The presence of B(a)P has significantly affected both the quantity of cyanobacteria and microalgae cells as well as their chlorophyll *a* (chl *a*) content and their ability to fluorescence. For many cyanobacteria and microalgae, an increase in cell numbers was observed after the addition of B(a)P. Therefore, even slight air pollution with benzo(a)pyrene is likely to facilitate the growth of airborne cyanobacteria and microalgae. The results provided an assessment of the organisms that are most susceptible to cellular stress following exposure to benzo(a)pyrene, as well as the potential consequences for the environment. Additionally, the results indicated that green algae have the greatest potential for degrading PAHs, making their use a promising bioremediation approach. *Kirchneriella* sp. demonstrated the highest average degradation of B(a)P, with the above-mentioned research indicating it can even degrade up to 80% of B(a)P. The other studied green algae exhibited a lower, yet still significant, B(a)P degradation rate exceeding 50% when compared to cyanobacteria and diatoms.

## 1. Introduction

Nowadays, air quality is one of the most critical issues concerning human health. The reduction of air pollution, caused by both chemical substances and microbial particles, represents a significant challenge today. Dealing with smog and viruses has become a particularly important issue in recent years, both in Europe and worldwide. A plethora of the literature indicates that in addition to chemical pollutants, bioaerosols, which encompass bacteria, viruses, fungi, pollen, cyanobacteria, and microalgae, negatively affect public health [1,2,3,4]. Studies on COVID-19’s spread since 2020, as well as previous reports on avian flu, measles, and SARS, have further demonstrated that viruses and bacteria pose a more significant threat to human health when present in polluted air [5,6,7,8,9,10,11].

The question arises whether cyanobacteria and microalgae can also pose a threat when present in poor-quality air. Compared to other bioaerosols, they are still insufficiently understood. In our present research, we focused on cyanobacteria and microalgae present in the air of an urbanized coastal zone. Cyanobacteria and microalgae were present in the air throughout the year, and their average abundance was similar to that of pollen in the atmosphere [12]. Among this group of bioaerosols, representatives of cyanobacteria were most frequently found in the atmosphere, and numerous green algae were also detected. Additionally, the presence of species belonging to Bacillariophyta, Charophyta, Haptophyta, Miozoa, Rhodophyta, and Ochrophyta phyla was observed [12]. Other researchers have also highlighted the abundance of cyanobacteria and microalgae in the atmosphere [13,14,15,16,17,18,19,20], which is extensively described in the work of Wiśniewska and colleagues [3].

In an urbanized coastal zone, cyanobacteria and microalgae coexist with chemical pollutants, including polycyclic aromatic hydrocarbons (PAHs), which are known to be human carcinogens and mutagens and toxic to other living organisms [21].

PAHs mainly originate from anthropogenic sources, including domestic, mobile, industrial, and agricultural activities [22]. The major sources of PAH emissions are coal and wood burning, petrol and diesel oil combustion, and industrial processes [22,23,24]. The European Community and the U.S. Environmental Protection Agency have identified PAHs as priority pollutants due to their negative impact on human health. Exposure to PAHs can lead to respiratory problems, such as impaired pulmonary function and bronchitis [25]. However, the health effects of individual PAHs vary depending on the structure of the molecule and the number of aromatic rings [22].

The most well-known PAH is benzo(a)pyrene (B(a)P), which is listed as a Group 1 carcinogen by the International Agency for Research on Cancer (IARC) [26]. B(a)P serves as a major indicator of PAH pollution, and its concentration in the air is regulated by the World Health Organization (WHO) and air quality agencies in Europe, North America, China, and several other countries [22,26]. The acceptable annual concentration for B(a)P in PM10 in European countries is 1 ng m^−3^ (Directive 2004/107/WE); however, this value is significantly exceeded, and the highest values are noted in the winter season [3].

It has been well established that microorganisms can transform polycyclic aromatic hydrocarbons (PAHs) [27,28]. According to Ghosal et al. [27], PAHs can be neutralized through various processes such as adsorption, volatilization, photolysis, and chemical oxidation. Among these methods, biotransformation by microorganisms is considered the most environmentally friendly approach to neutralizing high concentrations of PAHs in the atmosphere. Although the role of cyanobacteria and microalgae in PAH transformation is less understood compared to that of bacteria and fungi, recent scientific studies suggest that algae may play a crucial role in the degradation of PAHs. Warshawsky and his co-authors [29] demonstrated that the rate of degradation depends on several factors, including the amount of light energy emitted and absorbed, the number of PAHs exposed to the algae, the phototoxicity of PAHs and their metabolites, as well as the species and strain of algae. A review by Alegbeleye et al. [28] highlights the ability of different algae to transform various PAHs. The literature reports demonstrate that certain species of algae are capable of transforming PAHs such as naphthalene, phenanthrene, acenaphthene, pyrene, benz(a)anthracene, benzo(b)fluoranthene, benzo(k)fluoranthene, dibenzo(a, h)anthracene, indeno(1, 2, 3-c, d)pyrene, benzo(g, h, i)perylene, and benzo(a)pyrene [29,30,31,32,33,34,35,36]. A review also notes that even the highly toxic B(a)P can be transformed by marine algae into diols and quinones within 5–6 days [28]. However, it is important to consider whether the newly formed compounds are also toxic. According to Warshawsky et al. [29], several quinones, such as menadione, danthron, phenanthrene-quinone, and hydroquinone, were found to be highly phototoxic, whereas others, such as menadione, danthron, phenanthrene-quinone, and hydroquinone, were not.

The objective of this study was to investigate the interaction between airborne cyanobacteria and microalgae with polycyclic aromatic hydrocarbons (PAHs) using benzo(a)pyrene (B(a)P) as a model compound. In addition, we aimed to determine whether B(a)P has any effect on the life functions of cyanobacteria and microalgae and to identify the conditions that are conducive to such interactions. As this is the first scientific article devoted to the relationship between B(a)P and microalgae and cyanobacteria present in the air, we also suggest further necessary research directions.

## 2. Materials and Methods

### 2.1. Criteria for Selecting Experimental Organisms from Airborne Microalgae and Cyanobacteria

Microalgae and cyanobacteria were collected from a research station located 1 km from the coastal zone of the Gulf of Gdańsk in Gdynia, a region in the southern Baltic Sea, between 2018 and 2020 [12,19,37]. During 2018 and 2019, sampling was conducted during selected seasons, whereas in 2020, samples were collected throughout the year. The cyanobacteria and microalgae that were cultured, identified, and isolated for monocultures have been included in the Culture Collection of Baltic Algae (Airborne Algae—AA) at the Institute of Oceanography, University of Gdańsk, Poland [12,38]. A comprehensive list of the species isolated during the study period can be found in Wiśniewska et al. [38]. It is worth noting that not every detected taxon may have been isolated into a monoculture.

A study conducted in 2020 [12] revealed that cyanobacteria accounted for over 60% of all strains detected in the air, and Chlorophyta represented approximately 34% of them. The experiments were conducted on airborne cyanobacterial strains: *Nostoc* sp., (CCAA 03), *Synechococcus* sp., (CCAA 14), *Aphanothece* sp. (CCAA 48); green algae strains: *Oocystis* sp. (CCAA 20), *Kirchneriella* sp. (CCAA 38), *Coccomyxa* sp. (CCAA 21); and diatom strains: *Amphora* sp. (CCAA 34), *Halamphora* sp. (CCAA 47); *Nitzschia* sp. (CCAA 17). We utilized organisms from identical strains for our previous study, which investigated the influence of abiotic factors on the abundance and photosynthetic performance of these organisms [38]. The criteria for choosing these organisms included their prevalence in the air over the Baltic Sea area. A global analysis of bioaerosol availability reveals that cyanobacteria, green algae, and diatoms are among the most commonly found. Additionally, the chosen taxa were the most abundant among the other isolated taxa present in the coastal region of the Polish Baltic Sea [38].

### 2.2. Experimental Investigation of B(a)P Effects on Cyanobacteria and Microalgae in Laboratory Cultures

For the batch cultures, we utilized 25 mL glass Erlenmeyer flasks that contained sterilized F/2 medium [39]. These strains were cultured under a 16:8 h light: dark cycle, with photosynthetically active radiation (PAR) irradiance of 100 µmol photons m^−2^ s^−1^, and at various temperatures (10 °C, 15 °C, 20 °C, 25 °C, and 30 °C) while maintaining salinity at 8 PSU, which is representative of the average salinity in the Baltic Sea. We determined PAR levels using a Li-Cor (Lincoln, NE, USA) LI-189 model with a cosine collector, and salinity was determined using a salinometer (inoLab Cond Level 1, Weilheim in Oberbayern, Germany). The experimental setup was performed within thermostatic chambers (Biogenet, fitotron chamber, Józefów, Poland) that provided the necessary control over temperature conditions (±1 °C). The chosen temperatures were determined based on the varying temperature conditions observed within the Gdańsk Bay region. Specifically, the warm winters or early springs were favorable for the year-round presence of airborne algae, whereas the hot summers provide ideal conditions for algae blooms in the sea [12,37].

To prepare the cultures of isolated airborne cyanobacteria and microalgae for the experiments, they were first acclimatized to the new incubation conditions that corresponded to the proper culture conditions. The acclimatization period lasted for 7 days. After acclimatization, proper cultures of 20 mL volume were prepared. These cultures were in the logarithmic growth phase and contained a known number of initial cells. To prepare the cultures, a specific volume of inoculum (between 0.6 and 1.2 mL) was taken from the actively growing acclimatization culture (V = 20 mL) and added to a sterile F/2 medium. The number of initial cells in the proper culture was set at 10^5^ cells in 1 mL of the medium.

In this experiment, a specific concentration of benzo[a]pyrene (B(a)P) from Sigma-Aldrich was added to all strains, with concentrations in the sample ranging from 7.8 to 624 ng mL^−1^ (7.8, 15, 78, 312, and 624 ng mL^−1^). The test was performed with 3 replicates. These values were chosen to correspond to B(a)P concentrations in atmospheric particulate matter over the Gulf of Gdansk, ranging from 0.5 to 40 ng m^−3^ (0.5, 1, 5, 20, 40 ng m^−3^), based on previous monitoring of B(a)P concentration in the air over Gdynia (Gulf of Gdansk, Poland) [40,41,42,43,44]. The selected concentrations covered a range from low values to values well above the permissible annual average value for B(a)P in PM10 in EU countries, which is 1 ng m^−3^ (Directive 2004/107/WE). The test cultures were grown in incubator for one week until they reached the exponential growth phase, at which point the cell concentration, chlorophyll *a* content, photosynthesis performance, and B(a)P concentration were measured. Additionally, numerous blank samples were included, including analysis of a clean filter, a filter exposed only to B(a)P, and a filter exposed only to cyanobacteria and microalgae without the addition of B(a)P.

### 2.3. Calculation of Cell Density for Cyanobacteria and Microalgae

The quantification of cell numbers was performed using two different methods. The first method, described by Śliwińska-Wilczewska et al. [45], utilized a BD Accuri C6 Plus flow cytometer (BD Biosciences, San Jose, CA, USA) and was based on the linear correlation between cell concentration (N mL^−1^) and optical density (OD_750_). The second method, described by Śliwińska-Wilczewska and Latała [46], involved using a light microscope (Nikon Eclipse 80i, Nikon, Tokyo, Japan) and the Bürker counting chamber to quantify filamentous cyanobacteria. Both methods allowed for the determination of correlation coefficients and linear correlations between cell number and optical density.

### 2.4. Determination of Chlorophyll a

The concentration of chlorophyll *a* (chl *a*) in the tested cyanobacterial and microalgal strains was quantified according to established protocols [47,48]. Briefly, after 7 days of incubation, a 5 mL culture sample was filtered using a 0.45 µm filter (Macherey-Nagel MN GF-5, Dueren, Germany) and extracted using cold 90% acetone in the dark for 2 h at −20 °C. After centrifugation at 12,000× *g* rpm for 2 min (Sigma 2-16P, Osterode am Harz, Germany) to remove cell debris and filter particles, the extinction was measured using a UV-VIS Multiskan GO spectrophotometer (Thermo Scientific, Waltham, MA, USA) at 750, 665, and 480 nm with a 1 cm glass cuvette.

### 2.5. Determination of the Chlorophyll a Fluorescence in Cyanobacteria and Microalgae

The measurement of chlorophyll fluorescence was conducted using a pulse amplitude modulated (PAM) fluorometer (FMS1, Hansatech, King’s Lynn, United Kingdom). After 7 days of the experiment, the fluorescence parameter *F*_v_/*F*_m_ was analyzed. The 594 nm amber modulating beam with 4-step frequency control was used to provide illumination. The analyzed material was placed in the leaf clip on the 13 mm glass fiber filter (Whatman GF/C, Saint Louis, MO, USA). Saturating pulses of 0.7 s duration with an intensity of 4500 µmol photons m^−2^ s^−1^ were used to test airborne cyanobacterial and microalgal species. All samples were dark-adapted before the measurements.

### 2.6. B(a)P Analysis Using High-Performance Liquid Chromatography

To summarize, after 7 days of incubation, the cultures were filtered, and the B(a)P was isolated from the samples using solvent extraction with acetonitrile: dichloromethane (3:1 *v*/*v*) in an ultrasonic bath. The concentration of B(a)P was determined using high-performance liquid chromatography (Dionex UltiMate 3000) with a fluorescence detector (benzo(a)pyrene λex. = 296 nm, λem. = 408 nm). The chromatographic separation process was performed under gradient conditions using a mobile phase (water: acetonitrile) with a Thermo Scientific HYPERSIL GOLD C18 PAH chromatographic column (250 × 4.6 mm; 5 μm).

The solvents used for analyses were produced by Merc and were of HPLC grade. The benzo(a)pirene standard produced by Sigma-Aldrich (1000 μg/mL) was used to prepare calibration curves of the following concentrations: 0.1–10 ng·cm^−3^. The standard solutions for calibration curves were prepared in methanol. The linearity of the method was >0.999%. However, the precision, expressed as a coefficient of variation, was less than 15%. The limit of quantification of the method (LoQ) was defined as the 10-fold signal-to-noise ratio for a sample with a very low (close to the detection limit) content of B(a)P and was 0.01 ng cm^−3^. The recovery rate was 83% when compared to the reference material (SRM-2585). This procedure was previously used for determining the presence of B(a)P in the air. Nine strains were tested under several B(a)P concentrations (7.8, 15, 78, 312, and 624 ng L^−1^) and several temperatures (10 °C, 15 °C, 20 °C, 25 °C, and 30 °C), as well as additional blank samples.

### 2.7. Statistical Analyses

To investigate the impact of B(a)P concentration and temperature on the growth, chlorophyll *a*, and fluorescence of airborne cyanobacteria and microalgae, we utilized a repeated measures ANOVA. Significant differences between the control and treatment levels were determined through a post-hoc Tukey’s HSD test. Our data are reported as means ± standard deviations (SD), with statistical significance denoted by * *p* < 0.05. Prior to conducting these tests, normality and homoscedasticity were verified. Python [49] was employed to perform all statistical analyses.

## 3. Results

### 3.1. Variability in the Number of Cyanobacterial and Microalgal Cells

The experimental results indicated that the number of cyanobacterial and microalgal cells increased with the rise in ambient temperature despite the addition of benzo(a)pyrene (B(a)P, (ANOVA, *p* < 0.05) (Figure S1 in Supplement). A linear regression was performed to determine the average cell count values (based on the added concentration of B(a)P). The regression coefficient was found to be 0.95 for *Kirchneriella* sp., 0.96 for *Coccomyxa* sp., and 0.85 for *Oocystis* sp. For diatoms, the coefficient was 0.80 for *Nitzchia* sp., 0.93 for *Amphora* sp., and 0.73 for *Halamphora* sp. Meanwhile, for cyanobacteria, the coefficient was 0.64 for *Nostoc* sp., 0.96 for *Synechococcus* sp., and 0.86 for *Aphanothece* sp.

The average cell quantities for individual strains are presented in Table S1. For all tested organisms, the addition of a small amount of benzo(a)pyrene (increase in concentration from 0 to 7.8 ng mL^−1^) led to statistically significant changes in the number of cells (ANOVA, *p* < 0.05; Table S2).

**Figure 1:**
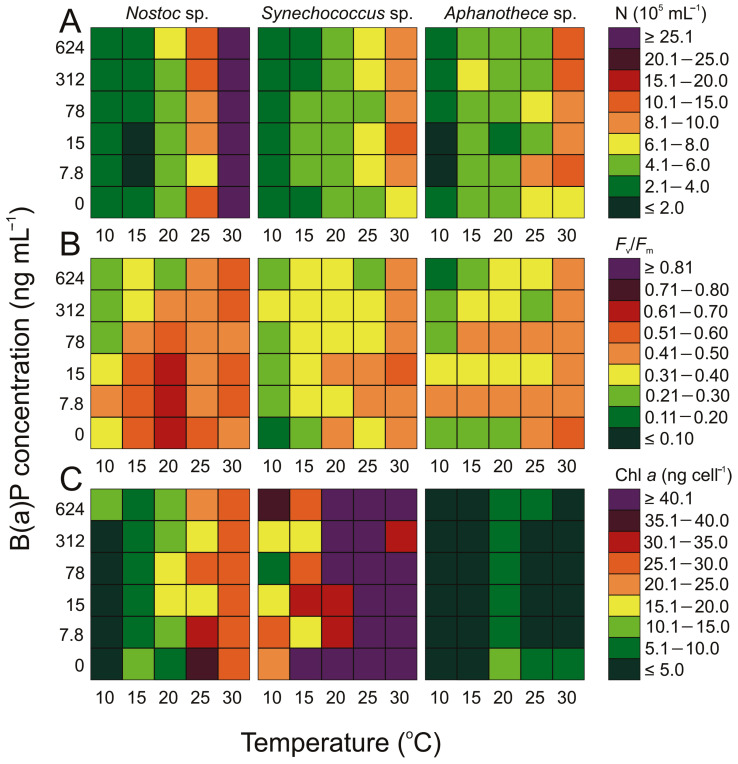
Changes in (**A**) the number of cells (10^5^ N mL^−1^), (**B**) maximum quantum efficiency of PSII photochemistry (*F*_v_/*F*_m_), and (**C**) chl *a* content (ng cell^−1^) for airborne cyanobacteria obtained after 7 days of experiment under different B(a)P concentration (ng mL^−1^) and temperature (°C) conditions.

Among the three tested cyanobacteria, *Nostoc* sp. showed the highest number of cells at a temperature of 30 °C: 46.05 × 10^5^ cell mL^−1^ (Figure 1). The addition of small concentrations of B(a)P (7.8 ng mL^−1^) at the highest temperature (30 °C) led to an increase in the number of cells of *Nostoc* sp., but a further increase in B(a)P concentration did not result in a linear increase in the number of cells. The minimum number of *Nostoc* sp. cells was 1.89 × 10^5^ cell mL^−1^ and was recorded at a temperature of 15 °C and a B(a)P concentration of 7.8 ng mL^−1^. For the remaining cyanobacteria, the highest cell numbers were observed at a temperature of 30 °C. The highest cell count for *Synechococcus* sp. was 1.01 × 10^5^ cell mL^−1^, which was observed at the highest temperature with a concentration of 15 ng mL^−1^ B(a)P, whereas for *Aphanothece* sp. it was 12.21 × 10^5^ cell mL^−1^ at a B(a)P concentration of 7.8 ng mL^−1^. The lowest values of 0.23 cell mL^−1^ for *Synechococcus* sp. were recorded at a B(a)P concentration of 15 ng mL^−1^ B(a)P and a temperature of 15 °C. In the case of *Aphanothece* sp., the lowest cell counts of 1.72 × 10^5^ cell mL^−1^ were observed at a B(a)P concentration of 7.8 and 15 ng mL^−1^ and a temperature of 10 °C. A linear relationship between the concentration of B(a)P and the number of cyanobacteria cells was not observed in the experiment.

**Figure 2:**
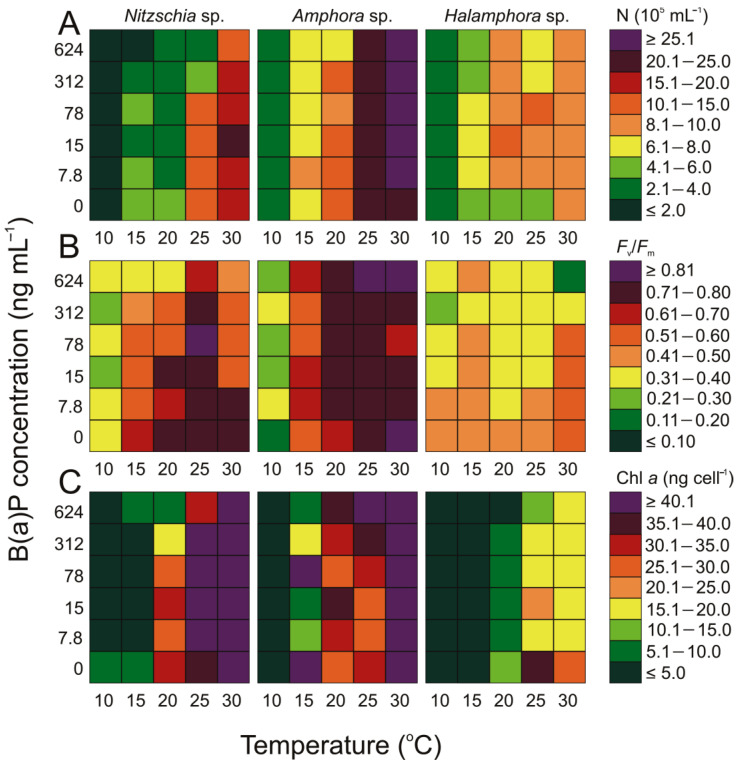
Changes in (**A**) the number of cells (10^5^ N mL^−1^), (**B**) maximum quantum efficiency of PSII photochemistry (*F*_v_/*F*_m_), and (**C**) chl *a* content (ng cell^−1^) for airborne diatoms obtained after 7 days of experiment under different B(a)P concentration (ng mL^−1^) and temperature (°C) conditions.

In addition to the cyanobacterium of *Nostoc* sp., a high number of cells was also found for the diatoms *Amphora* sp. (37 × 10^5^ cell mL^−1^) and *Nitzschia* sp. (approximately 22 × 10^5^ cell mL^−1^), which for both of them was achieved at a temperature of 30 °C and a concentration of 15 ng mL^−1^ B(a)P. On the other hand, the highest number of cells of *Halamphora* sp., 11.37 × 10^5^ cell mL^−1^, was observed at a B(a)P concentration of 7 ng mL^−1^ and a temperature of 20 °C, whereas the lowest number of cells, 2.61 × 10^5^ cell mL^−1^, was noted at a temperature of 10 °C without the addition of benzo(a)pyrene. Under similar conditions, the lowest number of *Nitzchia* sp. and *Amphora* sp. were recorded: 1.3 × 10^5^ cell mL^−1^ and 2.32 × 10^5^ cell mL^−1^, respectively. The addition of a low concentration of B(a)P (7.8 ng mL^−1^) resulted in a slight increase in the number of cells only at temperatures below 15 °C. However, a significant decrease in the number of organisms was observed at temperatures between 15 °C and 30 °C (Tukey HSD, *p* < 0.05; Figure 2). With an increase in the concentration of B(a)P, a decrease in the number of cells of both diatom species was noted, particularly evident in the case of *Nitzschia* sp. In the case of *Amphora* sp., similarly to cyanobacteria, the addition of a low concentration of B(a)P (7.8 ng mL^−1^) stimulated the growth of the tested organisms (ANOVA, *p* < 0.05). At the highest concentration of benzo(a)pyrene (624 ng L^−1^), however, the number of cells either decreased or remained at the same level as in the case of the B(a)P concentration of 312 ng L^−1^.

**Figure 3:**
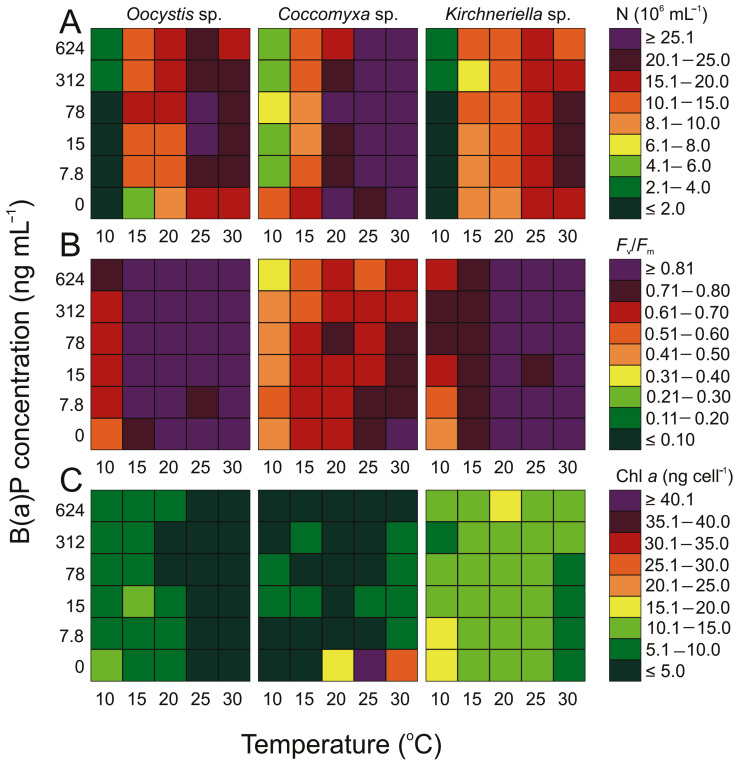
Changes in (**A**) the number of cells (10^5^ N mL^−1^), (**B**) maximum quantum efficiency of PSII photochemistry (*F*_v_/*F*_m_), and (**C**) chl *a* content (ng cell^−1^) for airborne green algae obtained after 7 days of experiment under different B(a)P concentration (ng mL^−1^) and temperature (°C) conditions.

The green algae selected for the experiment (*Oocystis* sp., *Coccomyxa* sp., *Kirchneriella* sp.) exhibited an increase in cell numbers upon the addition of B(a)P (ANOVA, *p* < 0.05). However, temperature was again the leading factor (ANOVA, *p* < 0.05). During the present study, the highest increase in Chlorophyta cell number was recorded after the addition of B(a)P at a concentration of 78 ng mL^−1^, which corresponds to a B(a)P concentration in the air of 5 ng m^−3^ (Figure 3). The highest number was demonstrated to be 73 × 10^5^ cell mL^−1^ for *Coccomyxa* sp., 24.08 × 10^5^ cell mL^−1^ for *Oocystis* sp., and 22.60 × 10^5^ cell mL^−1^ for *Kirchneriella* sp. The results obtained during the present study showed that the addition of B(a)P at higher concentrations, above 78 ng mL^−1^, resulted in a slight decrease in the cell number of green algae, especially noticeable at the highest temperature of 30 °C. On the other hand, the lowest values obtained for *Oocystis* sp. (0.4 × 10^5^ cell mL^−1^) and *Kirchneriella* sp (0.85 × 10^5^ cell mL^−1^) were achieved at a temperature of 10 °C with zero B(a)P concentration, whereas for *Coccomyxa* sp., it was at the lowest temperature but with a B(a)P concentration of 15 ng mL^−1^ (4.94 × 10^5^ cell mL^−1^).

### 3.2. Variability in Chlorophyll a Concentration

The concentration of chlorophyll *a*, recorded after a 7-day exposure to B(a)P and a control sample without B(a)P, is presented in Table S3 in the supplementary material. The significant fact is that the addition of a small amount of benzo(a)pyrene resulted in changes in the concentration of chlorophyll *a* (ANOVA, *p* < 0.05; Table S2).

*Synechococcus* sp. was characterized by the highest values of chlorophyll concentration among the studied cyanobacteria (with maximum 99 ng cell^−1^ at 7.8 ng mL^−1^ B(a)P and 30 °C; and minimum 5.21 ng cell^−1^ at 78 ng mL^−1^ B(a)P and 10 °C). The remaining cyanobacteria were characterized by lower concentrations of chlorophyll *a*. For *Nostoc* sp., the value of chl *a* ranged from 0.68 ng cell^−1^ (at 10 °C and no B(a)P) to 38.18 ng cell^−1^ (at 30 °C and no B(a)P), whereas for *Aphanothece* sp., the lowest value was 1.15 ng cell^−1^ (at 0 °C and 78 ng mL^−1^) and the highest value was 13.07 ng cell^−1^ (at 20 °C and no B(a)P). Interestingly, a decrease in the content of chl *a* was observed after the addition of B(a)P for the cyanobacteria *Aphanothece* sp.

Upon analyzing the concentration of chl *a*, we found that in some diatoms (*Nitzschia* sp. and *Halamphora* sp.), the concentration of chl *a* decreased with an increase in the concentration of B(a)P (Figure 2). The maximum chl *a* concentration for *Nitzchnia* sp. was 64.01 ng cell^−1^ (at 30 °C and 7.8 ng mL^−1^ B(a)P). Similarly to the other diatoms, at the lowest temperature, the amount of chlorophyll *a* was below the detection limit. On the other hand, for *Halamphora*, the highest value of chl *a* after adding B(a)P was 21.59 ng cell^−1^ (at 25 °C and 7.8 ng mL^−1^), which was much lower than the blank sample for *Hamaphora* at 25 °C (37.85 ng cell^−1^). The maximum amount of chl *a* for *Amphora* sp. was 70.47 ng cell^−1^. It was evident that the addition of a small amount of B(a)P (from 0 ng mL^−1^ to 7.8 ng mL^−1^) significantly reduced the chlorophyll *a* content for many diatoms, e.g., from 50.86 ng cell^−1^ to 11.02 ng cell^−1^ for *Amphora* sp. (at 15 °C).

*Coccomyxa* sp. had a low concentration of chl *a* and exhibited a decrease in chl *a* concentration values upon B(a)P addition, in comparison to the control samples. For example, at 25 °C the value decreased from 48.2 ng cell^−1^ to 3.95 ng cell^−1^. Furthermore, for this microalga, the lowest temperature recorded a chlorophyll concentration below the limit of quantification, whereas the highest concentration (8.63 ng cell^−1^) was found at the highest temperature at 78 ng mL^−1^ B(a)P. Other green algae are characterized by a higher content of chlorophyll *a* compared to *Coccomyxa* sp. after adding B(a)P. *Kirchneriella* sp. was characterized by the highest values of chl *a* concentration among the studied cyanobacteria after adding B(a)P (with maximum 16.24 ng cell^−1^ at 7.8 ng mL^−1^ B(a)P and 10 °C; and minimum 9.07 ng cell^−1^ at 15 ng mL^−1^ B(a)P and 30 °C). For *Oocystis* sp., maximum chl *a* concentration after adding B(a)P was 10.96 ng cell^−1^ at 15 °C and 15 ng mL^−1^, which was lower than maximum chl *a* for the blank sample with *Oocystis* sp. (13.23 ng cell^−1^ at 10 °C). The minimum value was 1.84 ng cell^−1^ at 30 °C and 624 ng mL^−1^. In the case of chl *a*, both temperature changes and also exposure to a small amount of B(a)P caused changes in the concentration parameter in the case of all strains (ANOVA, *p* < 0.05).

### 3.3. Variability in the Maximum Quantum Efficiency of PSII Photochemistry (F_v_/F_m_)

The mean values of the *F*_v_/*F*_m_ parameter before and after the addition of a small amount of B(a)P are presented in Table S4. Both the addition of benzo(a)pyrene and temperature changes significantly affect the value of the *F*_v_/*F*_m_ parameter (ANOVA, *p* < 0.05; Table S2).

The *F*_v_/*F*_m_ was low for the cyanobacteria. The lowest values were obtained in samples with the highest concentration of B(a)P. The maximum *F*_v_/*F*_m_ for *Nostoc* sp. was 0.65 at 20 °C and a B(a)P concentration of 7.8 ng mL^−1^, which was the same as the blank sample at this temperature. The remaining cyanobacteria were characterized by a lower *F*_v_/*F*_m_, which for *Synechococcus* sp. at its maximum was 0.52 at 15 ng mL^−1^ B(a)P and 30 °C, and for *Aphanothece* was 0.49.

In the case of diatoms, the maximum *F*_v_/*F*_m_ for *Nitzchia* sp. and *Amphora* sp. were similar, amounting to 0.81 at 25 °C and 78 ng mL^−1^, and 30 °C and 624 ng mL^−1^, respectively. For *Halamphora* sp., the maximum value was 0.58 at 30 °C and 15 ng mL^−1^. The minimum *F*_v_/*F*_m_ was similar to Cyanobacteria, which was 0.25, 0.22, and 0.19 for *Nitzchia* sp., *Amphora* sp., and *Halamphora* sp., respectively. The lowest value for *Nitzchia* sp. was noted at 10 °C and 312 ng mL^−1^, for *Amphora* sp. at 10 °C and 624 ng mL^−1^, and for *Halamphora* sp. at 30 °C and 624 ng mL^−1^. In the case of green algae, the obtained *F*_v_/*F*_m_ results differ significantly compared to the other studied bioaerosols.

The obtained maximum values amounted to 0.90, 0.79, and 0.89 for *Oocystis* sp., *Coccomyxa* sp., and *Kirchneriella* sp., whereas the minimum values were 0.64, 0.40, and 0.60, respectively. For the green algae, the maximum was noted at 30 °C and B(a)P 15 ng mL^−1^ for *Oocystis* sp., 7.8 ng mL^−1^ for *Coccomyxa* sp., and from 15 to 624 ng mL^−1^ for *Kirchneriella* sp. The minimum values for *Oocystis* sp. and *Kirchneriella* sp. were related at 10 °C and 7.8 ng mL^−1^, whereas for *Coccomyxa* sp. the minimum value was found at 10 °C and 624 ng mL^−1^. Both temperature changes and adding a small amount of B(a)P significantly affect the discussed parameter in the case of all strains (ANOVA, *p* < 0.05; Figure 3).

### 3.4. Variability in the Concentration of Benzo(a)pyrene after a 7-Day Exposure

**Figure 4:**
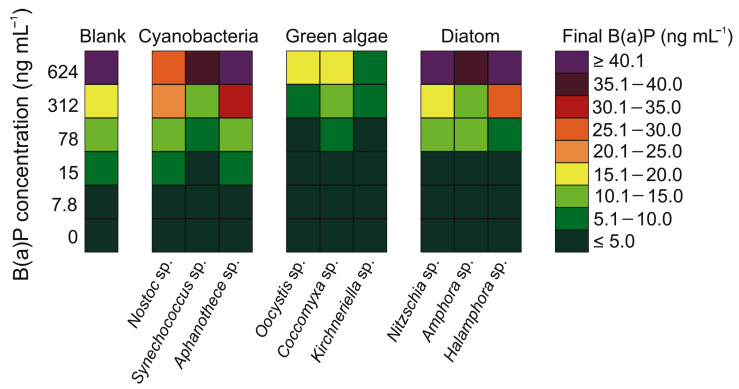
The final concentration of benzo(a)pyrene after 7 days of exposure for individual cyanobacteria and microalgae.

The results obtained during the experiment indicate that the concentration of benzo(a)pyrene was lower after seven days of exposure for each tested alga (ANOVA, *p* < 0.05; Figure 4, Table S5). For the purpose of this analysis, the concentrations obtained at individual temperatures were averaged. The reduction of the B(a)P concentration occurred also in the blank samples. Furthermore, the concentration of benzo(a)pyrene that remained in the samples after 7 days of exposure differed significantly between the green algae and the other groups (ANOVA, *p* < 0.05; Table S6).

Regarding diatoms, the highest mean B(a)P concentration was recorded in *Halamphora* sp. (20.1 ng mL^−1^), whereas the lowest mean concentration was observed in *Amphora* sp. (14.1 ng mL^−1^). In the cyanobacteria samples, the highest B(a)P concentration was noted in *Aphanothece* sp. (23.01 ng mL^−1^), whereas the lowest was observed in *Synechococcus* sp. (13.4 ng mL^−1^). In contrast, the concentration of B(a)P for green algae was two to three times lower than that of the other two algae species, with values of 7.23 ng mL^−1^, 3.3 ng mL^−1^, and 6.2 ng mL^−1^ for *Coccomyxa* sp., *Kirchneriella* sp., and *Oocystis* sp., respectively. The averaged values divided into individual B(a)P concentrations are presented in the table (Table S5). The results obtained during our seven-day experiment allowed us to establish that the average degradation of B(a)P was the highest for *Kirchneriella* sp. (80%). Additionally, *Oocystis* sp. was found to be responsible for 63% and *Coccomyxa* sp. for 56% of the B(a)P degradation.

## 4. Discussion

### 4.1. Cyanobacteria

The experimental results indicated that the number of cells of all three tested cyanobacteria increased significantly with increasing temperature regardless of the concentration of B(a)P (Figure 1). This is mainly related to the temperature preferences of specific organisms. Therefore, when conducting an experiment, it is worth focusing particularly on the analysis of results obtained under conditions preferred by a given organism. The addition of a small concentration of B(a)P, equivalent to 0.5 ng m^−3^ in the air, significantly affects the amount of microorganism cells, but it cannot be unequivocally stated that it always leads to growth. For *Synechococcus* sp. and *Aphanothece* sp., a slight stimulation of the increase in the number of cells was observed after the addition of B(a)P, especially at higher temperatures of 25 °C and 30 °C. The temperature is not random, especially considering the cyanobacteria, with particular emphasis on those occurring in the Baltic region, which prefer high temperatures, hence the most visible effects are observed at temperatures between 25 °C and 30 °C [12,50]. However, the increase in cell number was maintained regardless of a further increase in B(a)P concentration. The results obtained suggest that in the case of these organisms, small amounts of toxic substances can have a positive effect. On one hand, it can result in increased resistance and tolerance to stressors in certain organisms, which can be beneficial in environments with naturally occurring stressors or in areas with moderate levels of pollution. However, it can also lead to unexpected effects, such as promoting the growth of invasive species or causing the accumulation of pollutants in ecosystems.

In the case of *Nostoc* sp., the effect described above is not as pronounced as in the case of *Synechococcus* sp. and *Aphanothece* sp. The main difference between these cyanobacteria is the type of cell form. Both *Synechococcus* sp. and *Aphanothece* sp. have a microscopic size and a single-celled type, unlike filamentous *Nostoc* sp. However, the hypothesis that the cell form is the basis for differences in the occurrence of this process requires further detailed research.

Zhu and colleagues [51] have reported a similar phenomenon for *Microcystis aeruginosa*, another microscopic unicellular plankton. It appears that *M. aeruginosa* has a high tolerance to PAHs, and even low doses, this compound may have a positive effect on cell growth [51].

Moreover, in our experiment, we observed a decrease in the number of cells for each type of cyanobacteria at the lowest temperature (10 °C) after the addition of a small concentration of B(a)P (7.8 ng mL^−1^). This could be attributed to the low number of these organisms’ cells during the winter period in aerosols over the southern Baltic Sea region, when the air is known to be the most polluted with PAHs [12,43]. It also confirms the fact that the occurrence of specific cellular processes is closely related to the temperature preferences of organisms.

It is worth emphasizing that, even at concentrations as high as 624 ng mL^−1^, there was no significant decrease in the number of cyanobacteria cells. The ability of cyanobacteria to cope with PAHs may be due to their higher growth rate at higher temperatures. As one of the earliest organisms to populate the Earth, cyanobacteria have developed various defense mechanisms to survive in unfavorable environments. Some cyanobacteria are even capable of modulating the production of toxins in response to environmental stressors [51,52].

The maximum quantum efficiency of PSII photochemistry (*F*_v_/*F*_m_) is a widely used parameter to describe the physiological response of cyanobacteria and microalgae. It provides information on the efficiency of the light energy absorption and conversion into chemical energy during photosynthesis. The obtained research results indicate that the addition of a small amount of benzo(a)pyrene significantly affects the change in the *F*_v_/*F*_m_ parameter as well as the content of chlorophyll *a* in the cell, thus significantly affecting cellular stress. According to Zhang et al. [52], research on *M. aeruginosa* revealed that this organism possesses antioxidant defense enzymes such as superoxide dismutase (SOD) and catalase (CAT). The increased activity of these enzymes can provide protection against oxidative damage caused by PAHs. *M. aeruginosa* is known to produce a highly toxic compound called microcystin. However, studies have shown that this cyanobacterium can protect itself against oxidative stress caused by PAHs by stabilizing its photosynthetic apparatus and modulating protein metabolites [52,53,54]. As low values of *F*_v_/*F*_m_ are often observed when photosynthetic organisms are exposed to stress, indicating photoinhibition [55], the cyanobacteria were characterized by the lowest average value of this parameter, thus representing the group most susceptible to stress.

Based on our previous research, it was found that among the selected microorganisms isolated from the air, *Synechococcus* sp. showed the highest ability to produce microcystin [12]. Future research could shed light on the concentration of toxins produced by cyanobacteria in the air, especially because an increase in temperature can lead to the growth of cyanobacterial blooms and an increase in the number of cyanobacterial cells and their toxins in aerosols. This is particularly important because even a small concentration of B(a)P in the air, such as 0.5 ng m^−3^, could exacerbate the negative impact of airborne cyanobacteria on human health.

### 4.2. Ochrophyta

Among the analyzed microalgae, *Nitzschia* sp. and *Halamphora* sp. were found to be very sensitive to high concentrations of B(a)P. The growth inhibition observed in the diatom cells may suggest a lack of defense mechanisms against PAHs in these organisms at temperatures below 30 °C. Furthermore, lower concentrations of chl *a* were observed in the presence of B(a)P compared to the control sample. In the case of *Nizchia* sp. and *Halamphora* sp., the average ratio of *F*_v_/*F*_m_ was lower in the presence of B(a)P compared to the control sample, indicating increased cellular stress. Interestingly, the diatoms found in water tend to prefer lower temperatures for growth. There are diatoms, such as *Achnanthidium* sp. and *Fragilaria* sp., that have been found to have high growth rates between 10 °C and 30 °C [52]. The diatoms used in our experiment did not exhibit a significant increase in cell numbers at low temperatures, such as 10 °C.

The literature studies suggest that PAHs may have a particularly negative effect on diatoms. For instance, in the case of *Thalassiosira pseudonana*, they can impact the metabolism of fatty acids and the formation of silica shells [56]. Othman et al. [57] have reported that the presence of PAHs can impair the formation of diatom frustules, which in turn may lead to the inhibition of cell division and a decrease in growth rates. There are also studies that confirm the negative effect of B(a)P on photosynthesis in diatoms. Exposure to B(a)P can result in the downregulation of several proteins that are involved in photosynthesis [58].

On the other hand, the stimulation of an increase in the number of *Amphora* sp. at low concentrations of B(a)P across the entire temperature range may indicate the hormetic process, which is the phenomenon of a beneficial effect of small doses of a harmful factor on organisms. In the case of *Amphora* sp., despite the decrease in chl *a* concentration in the presence of B(a)P, the average values of *F*_v_/*F*_m_ compared to the control sample indicate that this organism slightly reduced cellular stress when in contact with B(a)P.

Diatoms are generally not commonly found in the atmosphere of the southern Baltic Sea region [3]. Their highest biomass in sea water is typically reached in the spring and winter, and their bloom typically starts at the turn of February and March [59]. At the same time, the highest concentrations of B(a)P are typically recorded in the air [40,42]. It is highly likely that the lack of defense mechanisms in diatoms against PAHs contributes to their low abundance in atmospheric aerosols. However, the reason for this can also be attributed to the larger size and weight of diatoms, resulting from their silica cell wall [3,12,59].

### 4.3. Chlorophyta

In general, green algae prefer to grow at temperatures between 27 °C and 32.8 °C [54]. Temperature is a factor that, on one hand, promotes organism growth. On the other hand, it may increase the toxicity of PAHs [57,60]. A study conducted by Vieira and Guilhermino [60] indicated that the toxicity of anthracene, naphthalene, and phenanthrene on *Tetraselmis chuii* increased with every 5 °C increase in temperature. In the case of green algae, regardless of the temperature, the number of cells as well as the concentration of chl *a* and *F*_v_/*F*_m_ changed under the influence of added B(a)P. Green algae differ from the microorganisms described above in many respects. Above all, they are the only microorganisms discussed here in which cellular stress decreased in the presence of B(a)P compared to the control sample.

Green algae appear to possess highly efficient defense mechanisms, as variations have been documented even among individual strains. In the case of *Kirchneriella* sp. and *Oocystis* sp., it can be observed that the concentration of B(a)P had a positive effect on the increase in the number of cells, but there was a limiting concentration beyond which the number of cells decreased. These organisms are characterized by a very high *F*_v_/*F*_m_, indicating an absence of cellular stress in the presence of B(a)P. The *F*_v_/*F*_m_ for *Kirchneriella* sp. and *Oocystis* sp. remained at a similar, constant value at temperatures above 10 °C, which suggests that these organisms were not stressed by the presence of B(a)P and were better able to tolerate PAHs even at higher concentrations (Figure 3). The difference in chl *a* concentration in the cells of these two green algae (*Kirchneriella* sp. and *Oocystis* sp.) and *Cocomyxa* sp. may be a key factor in the differences in their interaction with B(a)P. However, this hypothesis requires further investigation.

The results obtained indicate that Chlorophyta are the least sensitive of the analyzed algae to PAHs. Perhaps this is the reason why they are detected in the air of the coastal zone of the southern Baltic Sea throughout the year, even in winter when the concentration of B(a)P in the air is at its highest [12]. These organisms have a higher likelihood of occurring in the air during periods of high B(a)P concentrations compared to other cyanobacteria and microalgae, as observed in the southern Baltic Sea region [12]. However, our experiment indicated that all the green algae used in the experiment showed a reduction in the number of cells under the highest concentration of B(a)P (624 ng mL^−1^). At this point, it would be worth asking if there is a concentration limit of B(a)P that leads to this. It is worth considering whether this phenomenon is dependent on the air temperature or the amount of green algae bloom in seawater, or if there is a concentration limit of B(a)P that leads to this. Further research on this subject is advisable.

### 4.4. The Role of Microalgae and Cyanobacterial Cell in the Degradation of B(a)P

Reducing the amount of B(a)P in both control and other samples indicates that light, as a constant parameter in all samples, has an influence on the decomposition of the PAH itself. However, the presence of certain types of microorganisms may enhance this effect. According to Alegbeleye et al. [28], marine algae have been shown to transform B(a)P into diols and quinones, a process that can take between 5 and 6 days. However, Warshawsky et al. [29] pointed out that not all algae are able to exclude B(a)P and its phototoxic products through a physical barrier such as the cell wall, and that only certain algae have the appropriate dioxygenase enzymes to metabolize B(a)P into dihydrodiols and phototoxic products.

Scientists have indicated that green algae are capable of absorbing B(a)P and metabolizing it into dihydrodiols, whereas diatoms and cyanobacteria were unable to metabolize this PAH. Therefore, the obtained results indicate a statistically significant difference in the B(a)P content after 7 days of exposure between cyanobacteria and diatoms, as well as green algae. Figure 4 shows that the variability of B(a)P concentrations was very similar between diatoms and cyanobacteria. Thus, it was clear that green algae had the highest role in comparison to other bioaerosols. The differences between the amount of benzo(a)pyrene after a 7-day exposure between green algae and other types algae amounted to over 60%. However, the precise understanding processes that benzo(a)pyrene undergoes in the presence of cyanobacteria and diatoms require further intensive research to unequivocally confirm that these organisms do not degrade B(a)P.

The results obtained for the green algae indicate that *Kirchneriella* sp. has the highest ability to degrade benzo(a)pyrene. *Kirchneriella* sp. had the highest concentration of chlorophyll *a* and a high resistance to stress, which differed from other green algae. It should be acknowledged, however, that other green algae also have a high ability to degrade B(a)P. Green algae are abundant in chl *a*, which can absorb light energy for photosynthesis and is a major active substance that generates a high level of singlet oxygen. This singlet oxygen can catalyze the photo-transformation of B(a)P to quinones [35]. It may explain why *Kirchneriella* sp. had the highest efficiency in the removal of benzo(a)pyrene. The high degradability of B(a)P by green algae may favor their presence in the atmosphere throughout the year, including in winter when PAH emissions are at their highest. These organisms are recorded in Poland in the atmosphere also in the cold months when B(a)P concentrations in the air are exceeded. Thus, the above-described properties may favor their presence in the atmosphere. On the other hand, these studies indicate the potential use of green algae in air purification processes to remove this pollutant. A significant increase in the number of green algae cells in aerosols compared to other microorganisms may be responsible for the observed difference in B(a)P removal efficiency. Furthermore, scientific research confirms that even dead cells of green algae can effectively remove more than 90% of B(a)P [35]. In the presented studies, we did not assume the determination of the number of dead cells. However, in difficult weather conditions, a higher mortality of microorganisms than in water could favor the removal of pollutants. However, this hypothesis requires further research. It should be emphasized, however, that in our research, we did not observe the complete degradation of B(a)P by microalgae or cyanobacteria. Such a process is possible, but it is extremely rare [61].

## 5. Conclusions

Due to climate change, research on cyanobacteria and microalgae present in both sea and air has become increasingly important, as they play a significant role in the environment. Cyanobacteria and microalgae isolated from the air were subjected to B(a)P concentrations at varying temperature values. The addition of this compound, which is dangerous for humans, did not result in the complete death of any of the strains. On the contrary, many cyanobacteria and microalgae showed an increase in the number of cells after the addition of even small concentrations of B(a)P. The addition of benzo(a)pyrene caused significant changes in the cell number, concentration of chlorophyll *a*, and the maximum PSII quantum efficiency. The stimulation of growth of cyanobacteria and microalgae in the presence of low concentrations of benzo(a)pyrene can be significant in the context of biotechnology development and environmental protection.

Whether the given parameter decreased or increased depended individually on the strain, not the phylum. Both an increase in cell number and low cellular stress promote high B(a)P degradation. On the other hand, the concentration of chlorophyll *a* may be a key factor responsible for the differences in degradation between strains of green algae. Green algae proved to be a group with the greatest potential to degrade B(a)P, which may be considered as a promising bioremediation path. Of particular note is the green algae *Kirchneriella* sp., which stands out for its high B(a)P degradation among the studied bioaerosols. Low cellular stress and high degradability of pollutants may favor microorganisms inhabiting the atmosphere, especially during colder periods when B(a)P concentrations are at their highest.

## Data Availability

The data presented in this study are available on request from the corresponding author.

## Supplementary Materials

The following supporting information can be downloaded at: https://www.mdpi.com/article/10.3390/cells12071073/s1. Figure S1: variability of cyanobacteria and microalgae quantity with respect to sample incubation temperature in the presence of B(a)P (a) and without B(a)P (b); Table S1: the average, minimum, and maximum cell quantities for individual strains after 7 days of B(a)P addition and without B(a)P addition. Table S2: three-way factorial ANOVA of cell concentration, fluorescence, and pigment content measured in tested strains growing at different temperatures (°C) and B(a)P concentration (ng mL^−1^) in the range of 0 to 7.8 ng mL^−1^, df—degrees of freedom; F—Fisher’s F-test statistic; Mss—mean sum of squares and; Ss—sum of squares. Levels of significance were * *p* < 0.05; ** *p* < 0.01; *** *p* < 0.001. Table S3: the average, minimum, and maximum chlorophyll *a* for individual strains after 7 days of B(a)P addition and without B(a)P addition. Table S4: the average, minimum, and maximum *F*_v_/*F*_m_ for individual strains after 7 days of B(a)P addition and without B(a)P addition. Table S5: the average content of B(a)P [ng mL^−1^] after 7 days of exposure. Table S6: Two-way factorial ANOVA of B(a)P concentration after 7 days of exposure for tested taxa (divided as group of cyanobacteria, green algae, and diatoms), df—degrees of freedom; F—Fisher’s F-test statistic; Mss—mean sum of squares; and Ss—sum of squares. Levels of significance were * *p* < 0.05; ** *p* < 0.01; *** *p* < 0.001.
