# Supplementary material for: The Ability of Airborne Microalgae and Cyanobacteria to Survive and Transfer the Carcinogenic Benzo(a)pyrene in Coastal Regions
Source: Cells. 2023 Apr 2;12(7):1073. doi: 10.3390/cells12071073 (PMC10093748; doi:10.3390/cells12071073)
Supplement: Supplementary file 1 [file cells-12-01073-s001.zip › cells-2295852-supplementary.pdf]

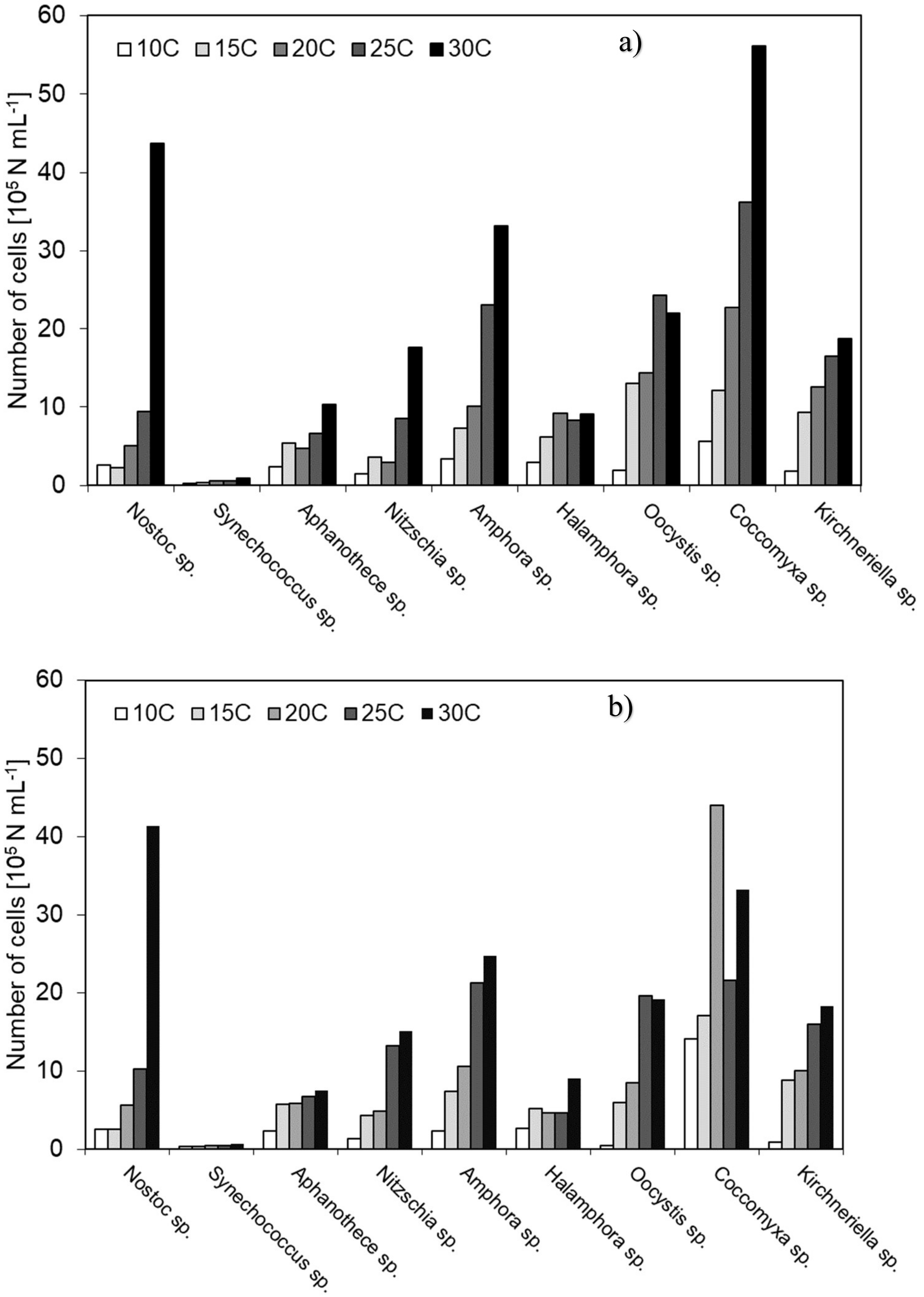

Fig. S1. Variability of cyanobacteria and microalgae quantity with respect to sample incubation temperature a) in the presence of B(a)P b) without B(a)P.

Table S1. The average, minimum and maximum cell quantities for individual strains after 7 days of B(a)P exposition and without B(a)P exposition.

	Nostoc sp.	Synechococcus sp.	*Aphanothece sp.*	*Nitzschia sp.*	*Amphora sp.*	*Halamphora sp.*	*Oocystis sp.*	*Coccomyxa sp.*	*Kirchneriella sp.*

Number of cells after adding B(a)P [·105 N x mL^{-1}]									
Av.	12.6	0.5	5.9	6.8	15.4	7.2	15.2	26.5	11.8
Min.	1.9	0.2	1.7	1.4	3.2	2.7	1.0	5.0	1.5
Max	46.1	1.0	12.2	22.0	37.4	10.4	27.2	73.0	22.6
Number of cells without B(a)P [·105 N x mL^{-1}]									
Av.	12.5	0.4	5.6	7.8	13.2	5.2	10.7	26.0	10.78
Min.	2.6	0.3	2.3	1.3	2.3	2.6	0.4	14.1	0.9
Max	41.4	0.7	7.5	15.1	24.7	9.1	19.6	44.0	18.2

Table S2. Three-way factorial ANOVA of cells concentration, fluorescence and pigment content measured in tested strains growing at different temperatures (0°C) and B(a)P concentration (ng mL^{-1}) in the range of 0 to 7.8 ng mL^{-1}: df – degrees of freedom; F – Fisher's F-test statistic; Mss – mean sum of squares; Ss – sum of squares. Levels of significance were: * $p < 0.05$; ** $p < 0.01$; *** $p < 0.001$.

	SS	Degr. of Freedom	MS	F	p
	Number of cells (cel mL^{-1})				
Intercept	8994.494	1.000	8994.494	20307.157	0.000***
Strain	5909.914	8.000	738.739	1667.875	0.000***
Temperature	4084.866	4.000	1021.216	2305.633	0.000***
B(a)P	20.138	1.000	20.138	45.467	0.000***
Strain*Temperature	7113.780	32.000	222.306	501.907	0.000***
Strain*B(a)P	45.220	8.000	5.653	12.762	0.000***
Temperature*B(a)P	26.366	4.000	6.591	14.882	0.000***
Strain*Temperature*B(a)P	130.340	32.000	4.073	9.196	0.000***
Error	79.726	180.000	0.443		
	Fluorescence (Fv/Fm)				
Intercept	93.778	1.000	93.778	35179.892	0.000***
Strain	5.818	8.000	0.727	272.799	0.000***
Temperature	2.352	4.000	0.588	220.598	0.000***
B(a)P	0.011	1.000	0.011	4.163	0.043*
Strain*Temperature	0.920	32.000	0.029	10.790	0.000***
Strain*B(a)P	0.072	8.000	0.009	3.360	0.001**
Temperature*B(a)P	0.070	4.000	0.017	6.546	0.000***
Strain*Temperature*B(a)P	0.170	32.000	0.005	1.990	0.003**
Error	0.480	180.000	0.003		
	Chl *a* (ng cel^{-1})				
Intercept	1444.490	1.000	1444.490	5981.596	0.000***
Strain	4721.586	8.000	590.198	2443.996	0.000***
Temperature	69.140	4.000	17.285	71.576	0.000***
B(a)P	0.768	1.000	0.768	3.181	0.076
Strain*Temperature	435.580	32.000	13.612	56.366	0.000***
Strain*B(a)P	1.163	8.000	0.145	0.602	0.775
Temperature*B(a)P	5.298	4.000	1.325	5.485	0.000***
Strain*Temperature*B(A)P	38.964	32.000	1.218	5.042	0.000***
Error	43.468	180	0.241		

Table S3. The average, minimum and maximum chlorophyll *a* for individual strains after 7 days of B(a)P exposition and without B(a)P exposition

	Nostoc sp.	Synechococcus sp.	*Aphanothece sp.*	*Nitzschia sp.*	*Amphora sp.*	*Halamphora sp.*	*Oocystis sp.*	*Coccomyxa sp.*	*Kirchneriella sp.*
Chl *a* after adding B(a)P [ng cell^{-1}]									
Av.	15.2	47.1	3.8	24.2	29.8	9.0	5.7	4.3	12.2
Min.	1.4	5.2	1.2	0	0	0	1.8	0	9.1
Max	38.2	98.8	13.1	64.0	70.5	37.9	11.0	8.23	16.2
Chl *a* cells without B(a)P [ng cell^{-1}]									
Av.	18.6	55.9	7.1	24.7	33.7	16.3	6.7	18.4	12.8
Min.	0.7	20.1	2.0	6.4	2.6	0	2.1	3.0	7.7
Max	38.2	94.0	19.1	41.7	55.0	37.9	13.2	48.2	16.5

Table S4. The average, minimum and maximum Fv/Fm for individual strains after 7 days of B(a)P exposition and without B(a)P exposition

	Nostoc sp.	Synechococcus sp.	*Aphanothece sp.*	*Nitzschia sp.*	*Amphora sp.*	*Halamphora sp.*	*Oocystis sp.*	*Coccomyxa sp.*	*Kirchneriella sp.*
Fv/Fm after adding B(a)P									
Av.	0.5	0.4	0.4	0.5	0.6	0.4	0.8	0.6	0.8
Min.	0.2	0.2	0.2	0.3	0.2	0.2	0.6	0.4	0.6
Max	0.6	0.5	0.5	0.8	0.8	0.6	0.9	0.8	0.9
Fv/Fm cells without B(a)P									
Av.	0.5	0.4	0.4	0.7	0.6	0.5	0.8	0.6	0.8
Min.	0.4	0.2	0.3	0.4	0.2	0.5	0.5	0.5	0.5
Max	0.6	0.5	0.5	0.8	0.8	0.5	0.9	0.7	0.9

Table S5. The average content of B(a)P [ng mL^{-1}] after 7 days of exposure

Added B(a)P [ng mL^{-1}]	*Nostoc* sp.	*Synechococcus* sp.	*Aphanothece* sp.	*Nitzschia* sp.	*Amphora* sp.	*Halamphora* sp.	*Oocystis* sp.	*Coccomyxa* sp.	*Kirchneriella* sp.	Blank sample
	B(a)P concentration after 7 days of exposition [ng mL^{-1}]									
7.8	3.6	3.0	1.4	4.1	2.1	1.3	2.2	1.6	1.5	2.1
15	9.5	3.2	5.1	2.3	2.3	1.9	0.9	0.9	2.0	6.5
78	10.8	9.5	10.5	10.6	13.8	7.9	3.2	5.6	1.1	10.0
312	22.3	14.2	30.1	15.8	14.9	29.2	6.9	10.6	6.2	19.0
624	25.9	37.1	74.8	43.8	37.5	70.1	17.8	17.6	5.6	48.2

Table S6. Two-way factorial ANOVA of B(a)P concentration after 7 days of exposure for tested taxa (divided as group of cyanobacteria, green algae, and diatoms): df – degrees of

freedom; F – Fisher's F-test statistic; Mss – mean sum of squares; Ss – sum of squares. Levels of significance were: * $p < 0.05$; ** $p < 0.01$; *** $p < 0.001$.

	SS	Degr. of Freedom	MS	F	p
	B(a)P concentration after 7 days of exposure (ng mL^{-1})				
Intercept	8076.261	1.000	8076.261	102.298	0.000***
B(A)P	7278.184	4.000	1819.546	23.047	0.000***
Strain	1368.927	2.000	684.463	8.670	0.001**
B(A)P*Strain	1528.676	8.000	191.085	2.420	0.038*
Error	2368.448	30.000	78.948		
